# Supplementary material for: Metformin is Associated with Reduced COVID-19 Severity in Patients with Prediabetes
Source: medRxiv. 2022 Aug 30:2022.08.29.22279355. Preprint. [Version 1] doi: 10.1101/2022.08.29.22279355 (PMC9460973; doi:10.1101/2022.08.29.22279355)
Supplement: 1 [file NIHPP2022.08.29.22279355V1-supplement-1.pdf]

medRxiv preprint doi: https://doi.org/10.1101/2022.08.29.22279355; this version posted August 30, 2022. The copyright holder for this preprint (which was not certified by peer review) is the author/funder, who has granted medRxiv a license to display the preprint in perpetuity.
This article is a US Government work. It is not subject to copyright under 17 USC 105 and is also made available for use under a CC0 license.

Supplemental Figures S1-S3

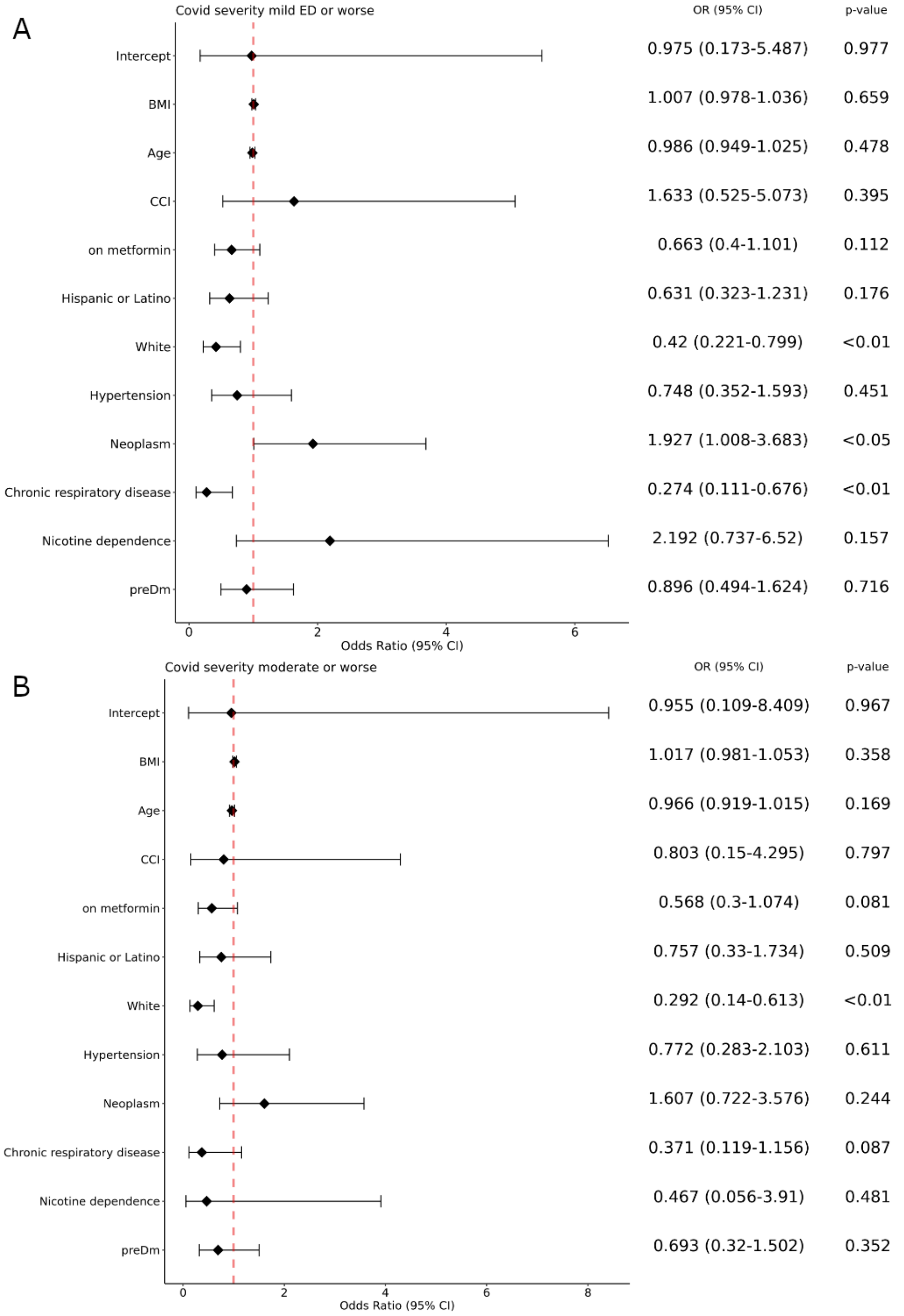

Figure S1. Forest plots for the PCOS cohort. A: Mild vs Mild ED and worse; B: Mild and Mild ED vs Moderate and worse.

medRxiv preprint doi: https://doi.org/10.1101/2022.08.29.22279355; this version posted August 30, 2022. The copyright holder for this preprint (which was not certified by peer review) is the author/funder, who has granted medRxiv a license to display the preprint in perpetuity.
This article is a US Government work. It is not subject to copyright under 17 USC 105 and is also made available for use under a CC0 license.

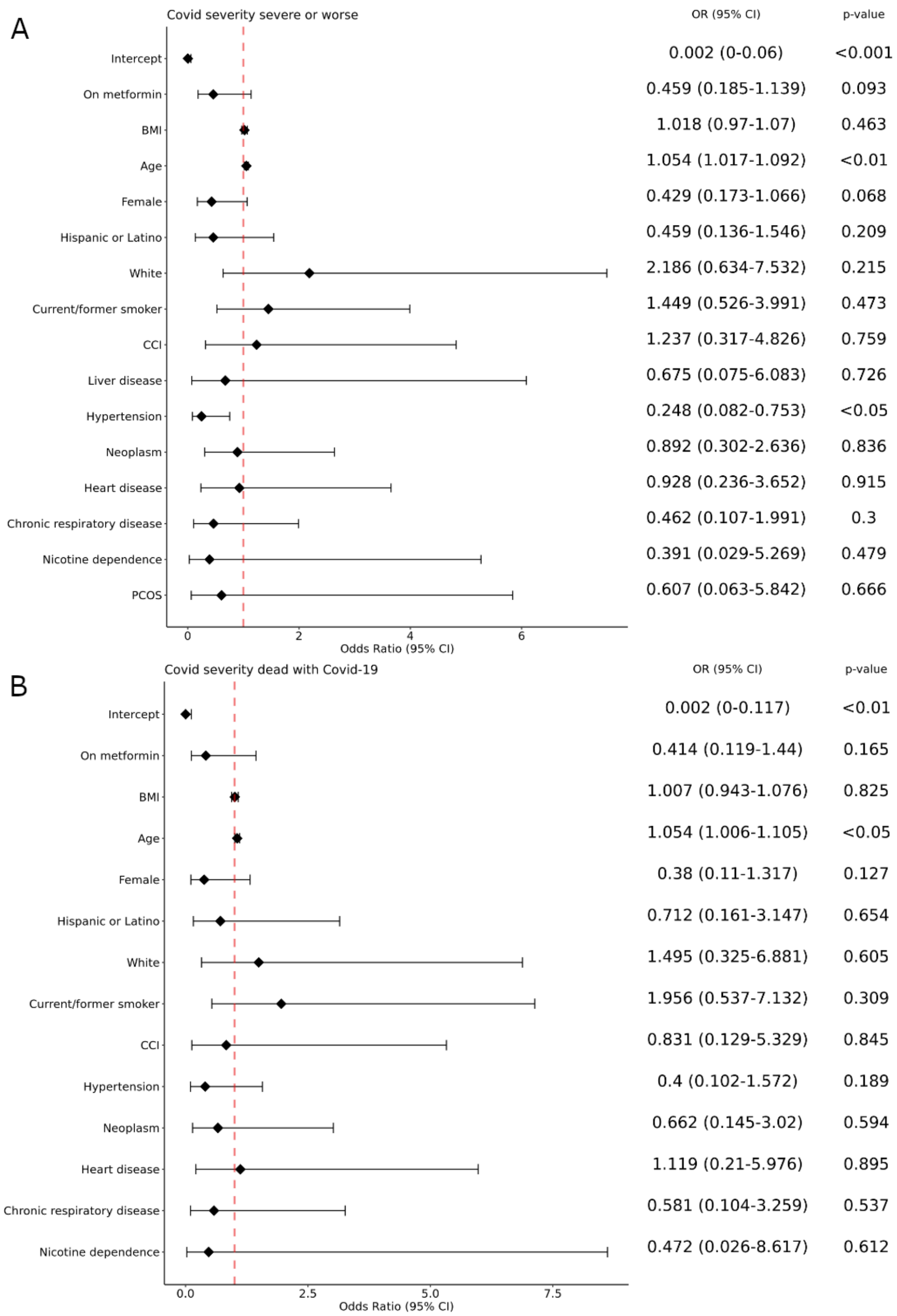

Supplementary Figure S2. Forest plots for the prediabetes cohort. A: Mild, mild ED, and moderate vs severe and worse, B: Mild, mild ED, moderate, and severe vs death with COVID-19

medRxiv preprint doi: https://doi.org/10.1101/2022.08.29.22279355; this version posted August 30, 2022. The copyright holder for this preprint (which was not certified by peer review) is the author/funder, who has granted medRxiv a license to display the preprint in perpetuity. This article is a US Government work. It is not subject to copyright under 17 USC 105 and is also made available for use under a CC0 license.

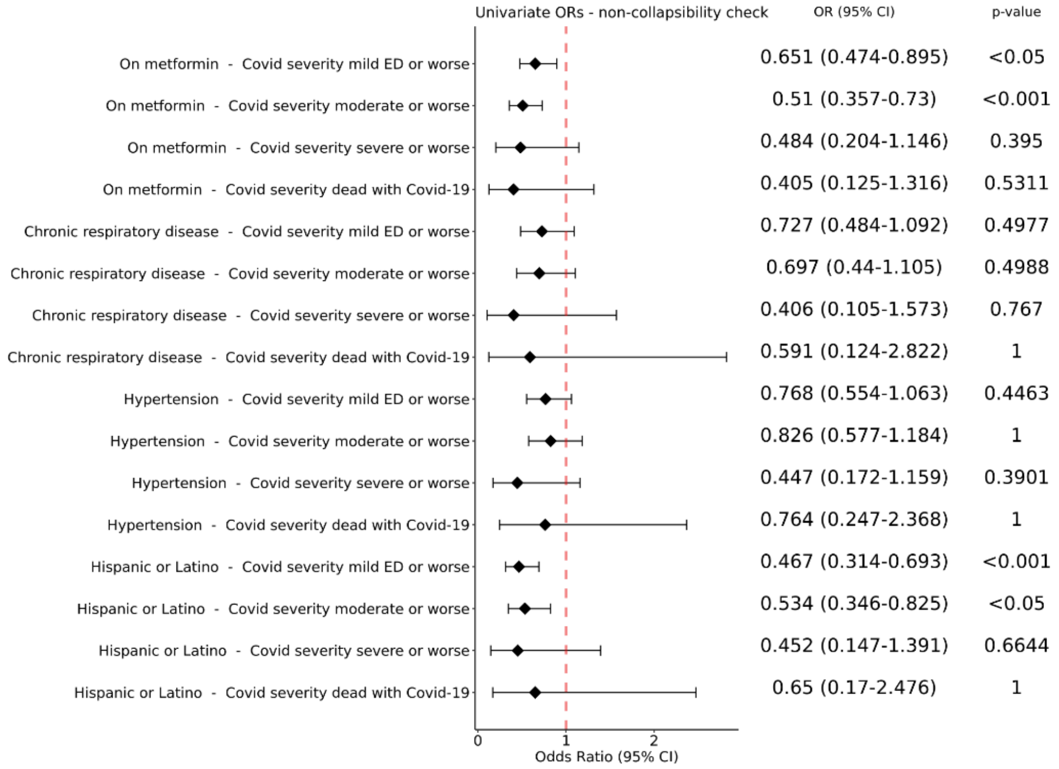

Supplementary Figure S3. Forest Plots for univariate odds ratios computed to assess the non-collapsibility effect for chronic respiratory disease. According to Schuster et al.,[50] non-collapsibility effects on a covariate can be estimated by comparing the OR obtained by univariate LR on the variable and the OR obtained in the multivariate analysis. To check whether the OR for chronic-respiratory disease is affected by the non-collapsibility effect we therefore run an univariate LR for each outcome. The forest plot reports the results obtained when univariate LRs were applied to either drug usage ("on metformin") or chronic respiratory disease; the ORs for drug usage are comparable to those obtained by multivariate LR (Fig. 4a, 4b) showing the reliability of the estimate. On the other hand, the remarkable difference in the ORs estimated for chronic respiratory disease confirm the non-collapsibility effect for this variable.
